# Characterization of alginate extracted from *Sargassum latifolium* and its use in *Chlorella vulgaris* growth promotion and riboflavin drug delivery

**DOI:** 10.1038/s41598-021-96202-0

**Published:** 2021-08-18

**Authors:** Shimaa R. Dalal, Mervat H. Hussein, Noura El-Ahmady El-Naggar, Sahar I. Mostafa, Sami A. Shaaban-Dessuuki

**Affiliations:** 1 Botany Department, Faculty of Science, Mansoura University, Mansoura, Egypt; 2 Department of Bioprocess Development, Genetic Engineering and Biotechnology Research Institute, City of Scientific Research and Technological Applications (SRTA-City), New Borg El-Arab City, Alexandria 21934 Egypt; 3 Chemistry Department, Faculty of Science, Mansoura University, Mansoura, Egypt

**Keywords:** Biomaterials, Polysaccharides

## Abstract

Alginates derived from macroalgae have been widely used in a variety of applications due to their stability, biodegradability and biocompatibility. Alginate was extracted from Egyptian *Sargassum latifolium* thallus yielding 17.5% w/w. The chemical composition of *S. latifolium* is rich in total sugars (41.08%) and uronic acids (47.4%); while, proteins, lipids and sulfates contents are 4.61, 1.13 and 0.09%, respectively. NMR, FTIR and TGA analyses were also performed. Crystallinity index (0.334) indicates alginate semicrystalline nature. Sodium alginate hydrolysate was evaluated as *Chlorella vulgaris* growth promoter. The highest stimulation (0.7 g/L biomass) was achieved by using 0.3 g/L alginate hydrolysate supplementation. The highest total soluble proteins and total carbohydrates were 179.22 mg/g dry wt and 620.33 mg/g dry wt, respectively. The highest total phenolics content (27.697 mg/g dry wt.), guaiacol peroxidase activity (2.899 µmol min^−1^ g^−1^) were recorded also to 0.3 g/L alginate hydrolysate supplementation. Riboflavin-entrapped barium alginate-Arabic gum polymeric matrix (beads) was formulated to achieve 89.15% optimum drug entrapment efficiency (EE%). All formulations exhibited prolonged riboflavin release over 120 min in simulated gastric fluid, followed Higuchi model (R^2^ = 0.962–0.887) and Korsmeyer–Peppas model with Fickian release (n ranges from 0.204 to 0.3885).

## Introduction

Marine algae are plant-like organisms that are typically found fixed on hard bases in coastal regions. They are oxygen producers, represent the food base for most of the aquatic life and represent the main source of crude oil, food, and many pharmaceutical and industrial products for humans^1^. Currently, natural macroalgal-derived polysaccharides have attained more attention owing to their distinctive characteristics as stability, biodegradability in addition to biocompatibility. Seaweeds represent a promising potential source of numerous bioactive compounds as a variety of polysaccharides, phenolic compounds, pigments, vitamins, and dietary fibers that have been experimentally assessed for their biological impact of newly produced medications^2^.

Alginate is a gelling anionic polysaccharide derived from phaeophytes. 1,4-linked-d-mannuronic (M residues) and L-guluronic acids (G residues) represent the constituents of the linear biopolymer alginate which are partitioned into homopolymeric blocks (G- and M-blocks) and heteropolymeric blocks (MG-blocks)^3^. The viscosity as well as gel strength of the algal polysaccharide solution are affected by the type of algal polysaccharide, temperature, pH, and presence of ions such as K^+^ and Ca^2+^^4^. Owing to pH sensitivity of the alginate (ionotropic gelation); it shrinks in an acidic medium and swells in high pH conditions^3^. Berg et al*.*^5^ was the first to apply organic gels in drug delivery systems, then, attention has increased to use these materials in the pharmaceutical industry^6^. García-González et al*.*^7^ manufactured alginate in the form of aerogels, microbeads of pectin and starch and applying them as drug carriers.

Alginate oligomers could be prepared by two methods. Enzymatic digestion by alginate lyase is the most common method, producing alginate oligomers with an unsaturated terminal construction. The second method is acid hydrolysis, which produce alginate oligomers with saturated terminal construction. Iwamoto et al*.*^8^ revealed that alginate oligomers terminal construction has a great impact on its biological reactions, they elucidated that alginate oligomers of terminal construction with double bonds exhibits greater activities than oligomers with saturated terminal construction. Oligomers are applied to promote a variety of biological and physiological activities of plants, such as seed germination, shoot elongation, root development, flower development, antimicrobial activity, heavy metal stress alleviation, phytoalexin activation^2^, microchlorophytes growth promotion^9^.

*Chlorella* species are very significant for the lipids and biomass production^10^, adaptable to various environmental conditions^11^, tolerant to high CO_2_ concentrations^12^, helpful in the treatment of industrial effluents^13^ and purification of waste-water systems^14^.

Alginate is amongst the most preferable biopolymeric substances used in drug delivery and encapsulation systems because it is biocompatible, biodegradable, available and affordable biopolymer^15^. Some bioactive constituents are incorporated within a matrix of certain formulations for attaining some activities as immobilization, protection, stabilization in addition to controlled release. Furthermore, alginate forms a thermally stable biocompatible hydrogel system in the presence of di-valent or tri-valent cations^16^. On the other hand, formation of alginate beads can be simply achieved by dropping of alginate solution into calcium chloride solution. The formed alginate beads are included in many capsulation applications such as biomedical, bioprocess, pharmaceutical fields in addition to food and feed industries^17^. Alginate applications might be significantly affected by its chemical composition, concentration, purity in addition to the valency and the concentration of the gelling cations.

Among the main innovation challenges nowadays is the encapsulation of bioactive molecules for drugs and food supplements and/or controlling their release system for improved drug bioavailability. These encapsulated hydrogel beads, after loading, can be mentioned as immobilized biocatalysts for the regulated release of the encapsulated molecules^18^. Being nontoxic, alginate is one of the most widely used polymers in drug delivery systems which deliver proteins and drugs, protecting them from destruction by gastric juice^19^. The foundation of encapsulation process is to produce a biocompatible barrier between the medium and the substrate loaded in the protective matrix^16^. Hydrogel matrices are cross-linked networks of natural molecules modified to attain organized release of biologically-active compounds and are affected by interaction with environmental stimuli^20^. These hydrogels are appropriate to encapsulate hydrophilic molecules to be capable of water holding. Riboflavin (vitamin B2) is one of the hydrophilic, water-soluble bioactive molecules, it has been selected as a model drug in many studies on encapsulation and organized sustained release of hydrophilic active compounds^20^.

The present study was designed to extract and characterize alginate derived from the Egyptian phaeophyte, *S. latifolium,* in addition to evaluation of *Chlorella vulgaris* growth criteria in response to sodium alginate hydrolysate supplementation, as well as assessing alginate blended with Arabic gum potentiality as a carrier matrix for riboflavin drug delivery in simulated gastric juice.

## Results and discussion

### Yield and chemical composition of the extracted sodium alginate

*Sargassum latifolium* yielded 17.5% (w/w) sodium alginate based on the dry biomass. Our results were in accordance with those obtained by Chandía et al*.*^21^ who reported that sodium alginate obtained from *Lessonia vadosa* was 3.0–17.7%/dry weight and Belattmania et al*.*^22^ who reported that sodium alginate yield obtained from *Sargassum filipendula* was17% (w/w). On the other hand, Mohammed et al*.*^23^ applied response surface to optimize the alginate extraction from *Sargassum* sp. and achieved 28% alginate yield.

Results demonstrated that 41.08% for total sugars, 47.46% for uronic acids in addition to minor contents of proteins (4.61%), lipids (1.13%), water (2%) and 0.09% for sulfates and low amounts of fucoidans were co-extracted from *S. latifolium*^24^*.* The present results are in agreement with the results obtained by Larsen et al*.*^25^, Duarte et al*.*^26^ and Gomaa et al*.*^27^ who documented that alginate and/or fucoidan are the main components of polysaccharide matrix in most *Sargassum* species. In accordance with our results, Viswanathan and Nallamuthu^28^ reported that protein and lipids contents of sodium alginate derived from some seaweeds were 1.4%—7.17% and 1.76%—6.12%, respectively.

### UV–visible analysis

UV–visible spectrogram of alginate extract (Supplementary Fig. S1) illustrated the assignment of absorption peak in 200–400 nm range recognizing the carboxylate and proteinaceous constituents as suggested by Osman et al*.*^29^. Peaks at 264 nm, 348 and 392 nm can be contributed to the presence of phenolics as well as flavonoids and their derivatives^30^. Data demonstrated absorption bands lies between 260 and 400 nm, which distinguish the presence of aromatic and poly-aromatic compounds comprising proteins and amino acids^31^.

### Proton nuclear magnetic resonance (^1^H NMR) analysis

**Figure 1:**
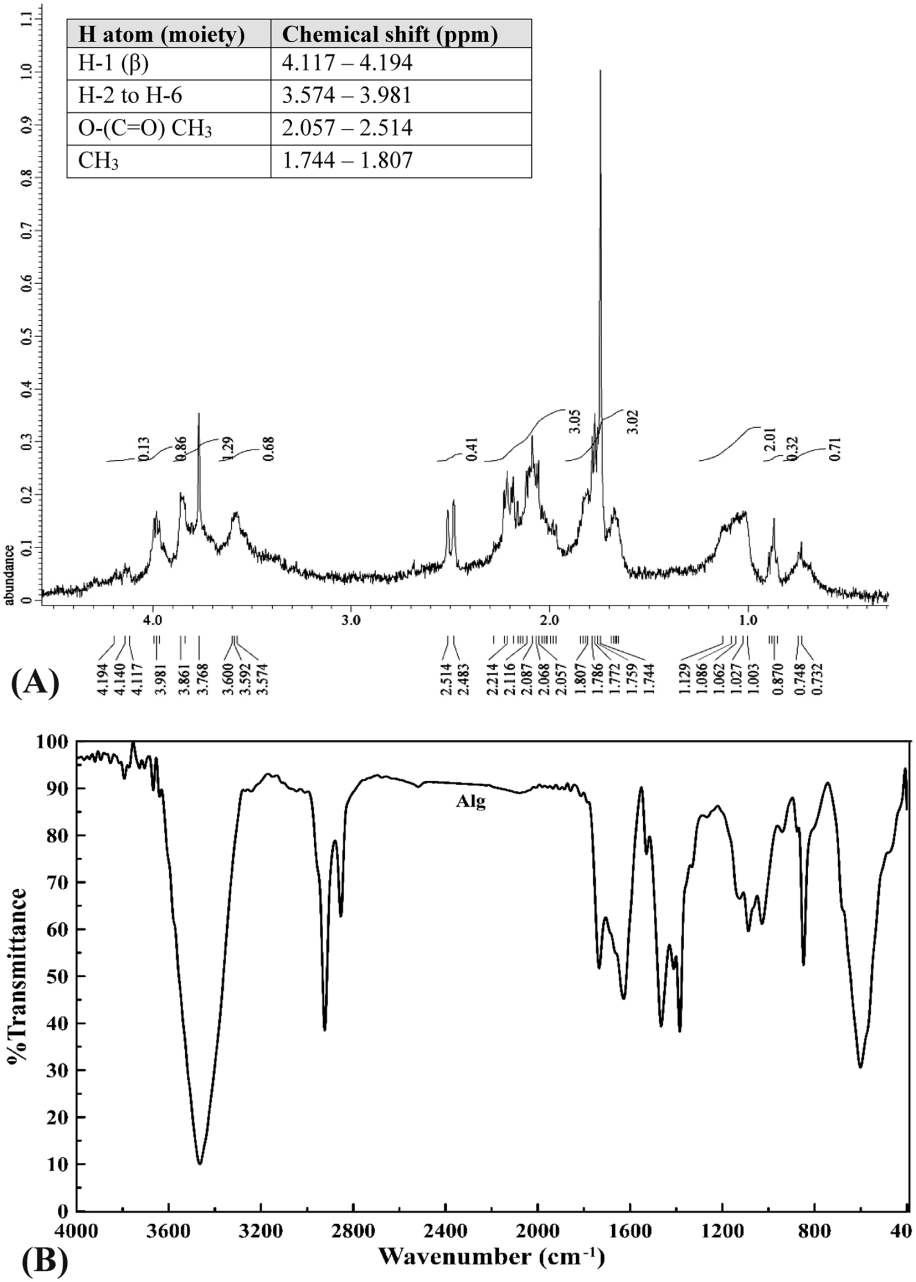
(**A**) NMR of extracted sodium alginate, (**B**) FTIR analysis of sodium alginate.

^1^H NMR spectroscopy is a significant physicochemical method for elucidating structure of polysaccharides. Structural features of alginate are elucidated by the ^1^H NMR profile (Fig. 1A) which depict the chemical shifts and single monomers and blocks characteristic to the sodium alginate fraction (Fig. 1A), revealing purity^32^. Data revealed the recognition of β-anomeric protons in the alginate sample in addition to appearance of protons signals within a 2-ppm chemical shift range (3 to 4 ppm). Assignments of the signs in the anomeric zone are recognized^33^ whereas, 2 signals in the anomeric region were documented.

The present results illustrated Signal II corresponding to the overlap between mannuronic acid anomeric proton (M-1) and the H-5 of alternating blocks (GM-5) besides, Signals III corresponding to proton H-5 guluronic acid from the GG-5G block (G-5) in the alginate sample indicating the heterogeneous (FGM) blocks of alginate^34^. The ^1^H NMR profile revealed presence of guluronic acid H-5 (GG-5G) that was recognized at 4.282 ppm (signal III) as suggested by Usoltseva et al.^32^ and Flórez-Fernández et al*.*^35^. Furthermore, the characteristic signals of fucose at 1.744–1.807 ppm for alginate revealed presence of neutral sugars traces of fucoidans which co-extracted from brown algae as interpreted by Bouissil et al*.*^24^. Moreover, the overlapping signals in the ^1^H NMR could be an assignment for the structure complexity of the alginate sample^34^. Aside from alginate extracted from *Sargassum vulgare* by Hussein et al*.*^31^, most *Sargassum*—derived alginate have M/G < 1 and significant magnitudes of homopolymeric block M (η < 1). The heterogeneity of alginate composition could be affected by the environmental factors^23^.

### FTIR Spectrum of sodium alginate

Sodium alginate FTIR profile (Fig. 1B, Supplementary Table S1) revealed different chemical functional groups. FTIR spectrogram of alginate is dominated with strong absorption broad band at 3465 cm^−1^ ascribing hydroxyl group (–OH stretching). Whereas, spectral peaks allocated in the range 4000 cm^−1^–3400 cm^−1^ could be assigned to alcohol and acids^35^. Strong peaks in the range of 1628 to 1428 cm^−1^ designated asymmetric and symmetric stretching vibrations that attributed to carboxylate anions (COO^−^)^35^. These significant spectral peaks could take a part in the structure elucidation of alginates recognizing the metal-carboxylate interactions according to Flórez-Fernández et al*.*^35^. The spectral band around 2923–2854 cm^−1^ is in agreement with that obtained by Aprilliza^36^ and are attributed to aliphatic –CH stretching, and symmetrical and asymmetrical (C–H)CH_2_ stretching, beside aromatic and/or vinylic C–H stretching and (CH)-anomer stretching^37^.

FTIR spectrogram of alginate also illustrated peak of C–H stretching vibrations recognizing alkanes, C=O indicated carbonyl group (amide I band), COO^−^ stretching vibrations ascribed to carboxylate as well as C–O–C stretching vibrations^36^. Peaks attained the range of 1090–1030 cm^−1^ are assigned to C–O stretching of pyranosyl ring, C–O–C asymmetric stretching (glycosidic linkage), C–C stretching which are attributed to alginate saccharide structure^38^. Also, the stretching of C = O group was documented at 1734 cm^−1^ as reported by Carpenter and Saharan^39^. The present results are in accordance with those of Cardenas-Jiron et al*.*^40^ and Bouissil et al*.*^24^. According to Gomaa et al*.*^27^, the spectral band 848 cm^−1^ confirms the presence of sulfate groups of fucoidan. Peaks around 600 cm^−1^ could be due to symmetric and asymmetric O=S=O deformation as reported by Flórez-Fernández et al*.*^35^.

### Thermogravimetric analysis (TGA)

**Figure 2:**
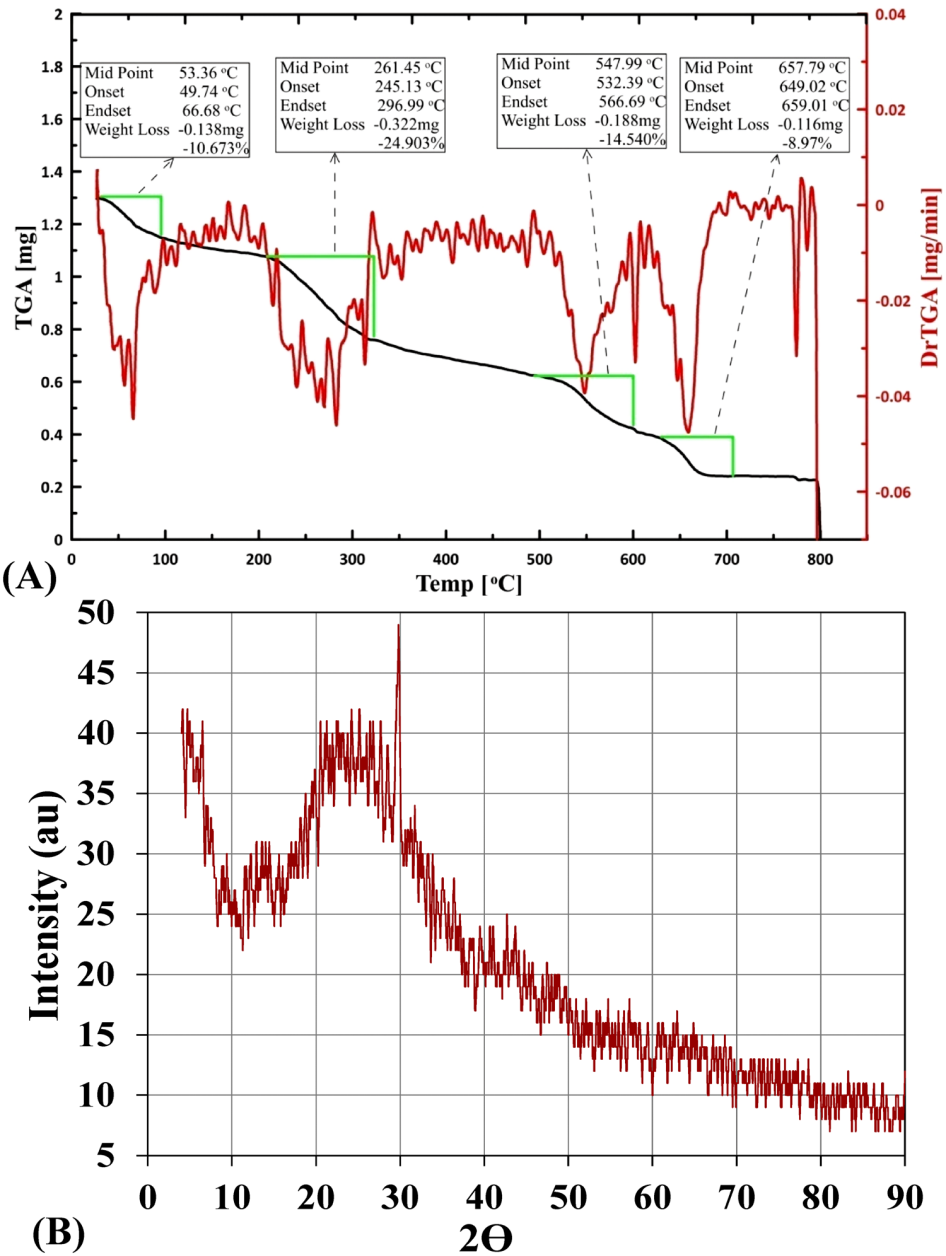
(**A**) Thermogravimetric analysis (TGA), (**B**) X-ray diffraction of extracted sodium alginate.

The thermal behavior of alginate (Fig. 2A) indicated mass progressive decreasing pattern with increasing temperature, demonstrating four distinctive decomposition stages characterized by temperature range for each of them. The first stage began with weight loss of 10.67% in the temperature range 49.74–66.68 °C, followed by a 24.90% mass loss in 245.13–296.99 °C temperature range. Thereafter, 11.54% mass loss has documented in the temperature range 532.39–566.69 °C, ended with 8.97% mass loss within temperature range 649.02–659.01 °C. Under progressive elevating temperature, sodium alginate exhibited initial dehydration process during the first stage. The initial dehydration followed by two decomposition stages characterized by the production of carbonaceous residue. The decomposition stages followed by production of sodium carbonate with 40.91% carbonized matter residue at the end of the experiment which is degraded gradually according to the interpretation of Guedes Soares et al*.*^41^, exhibiting good thermal stability. The present thermal degradation behavior of sodium alginate is in agreement with those of Rani et al*.*^42^ and dos Santos Araújo et al*.*^43^. On the other hand, Xu et al*.*^44^ reported the thermal stability of calcium alginate capsules up to 160 °C.

### X-ray diffraction (XRD)

The XRD pattern was used to investigate the micro structural features of *Sargassum latifolium*—derived sodium alginate (Fig. 2B). The XRD profile illustrates three distinctive intensive crystalline diffraction, which observed at 2θ degrees values of 20.51, 21.04 and 29.73 with inter planar spacing (d-spacing) of 4.328 Å, 4.22 Å and 3.003 Å, respectively indicating a rather amorphous structure. The obtained diffractogram demonstrate crystallinity index of 0.334 indicating the semi-crystalline nature of this sodium alginate sample^45^. Furthermore, owing to the interpretation of Kanimozhi et al*.*^46^ the peak height intensity and the degree of crystallinity could diagnose the amorphous nature of alginate. In accordance with our results, Aprilliza^36^ reported that sodium alginate extracted from brown algae has a semi-crystalline structure.

### Rheological measurement

**Figure 3:**
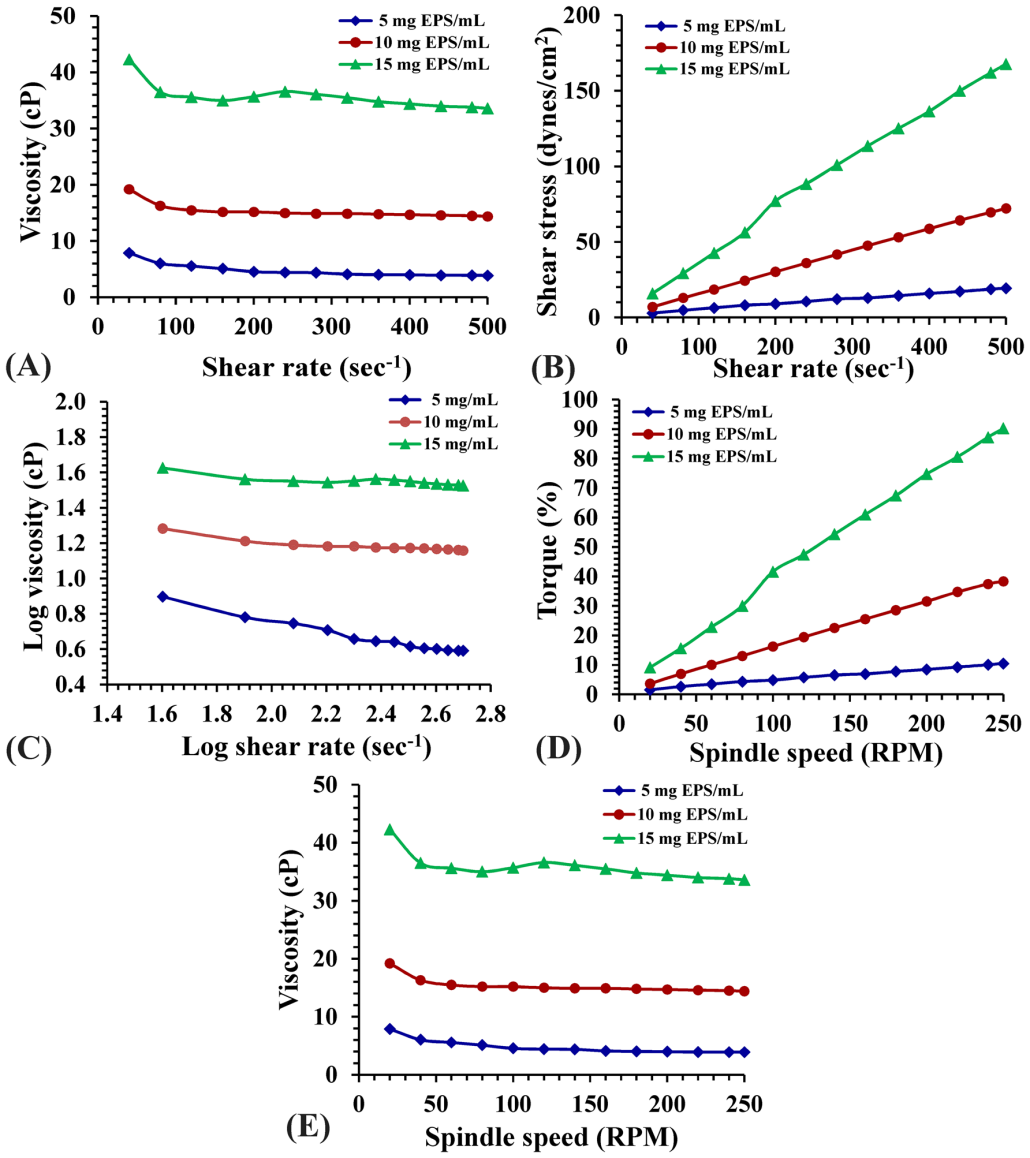
(**A**) Viscosity as a function of shear rate, (**B**) Flow curve of the shear stress vs. shear rate, (**C**) Log–log plot of the viscosity vs. shear rate, (**D**) Rheogram of the Torque vs. spindle speed and (**E**) Rheogram of the viscosity dependence of spindle speed (RPM) of aqueous solutions of extracted sodium alginate at concentrations 5, 10 and 15 mg Alg/mL.

Rheological characteristics are significant parameters for the biotechnology issue of phycocolloids. The apparent viscosity of sodium alginate fraction in aqueous solutions (5, 10 and 15 mg/mL) was quantified as a function of shear rate in the range 20–500 s^−1^ that is illustrated in Fig. 3. Flow profile demonstrated that 40 s^−1^ shear viscosity giving maximum values of viscosity 8.03, 12.1, 22.1 centipoise (cP); respectively (Fig. 3A). At the maximum shear rate used (500 s^−1^), the viscosity exhibit marked decrease in all alginate concentrations to 2.3, 5.1, 11.2 cP; respectively designating a shear thinning behavior^47^. Non-Newtonian flow is studied via gathering the viscosity data covering the range of shear rates used to perform a rheogram representing viscosity versus shear rate. Despite the suggestion of Wildemuth and Williams^48^ about the presence of conflicting issue of the distinctive shear-thinning fluids that displays an area of Newtonian flow at shear rate extremes, the present data did not illustrate any Newtonian flow behavior for the studied alginate solutions. According to the findings of Shyamali^49^, *Sargassum* species—derived alginates include a higher quantity of guluronic acid blocks which yielded strong gels relative to that derived from *Macrocystis*. The detected shear-thinning pseudoplastic performance, which is considered as an irreversible structural disturbance, and the reduction of viscosity takes place due to molecular arrangement which occurs in the structural rearrangement as indicated by Glicksman^50^. Flow curves of different concentrations of alginate (Fig. 3B) exhibited decreases in shear stresses showing a restrictive stable value at reduced values of shear rate designating limited yield stress of these solutions. Though greater yield stress levels could be reached with the high concentrations of alginate. The pseudoplastic property showed as shear thinning of alginate solutions is illustrated in Fig. 3C. The highest viscosity levels as well as the marked shear thinning behavior were documented to the highest concentration ending with the smallest mg alginate/mL. Rheogram (Fig. 3D) torque percent increases with increasing spindle speed (RPM). Figure 3E explains viscosity of alginate aqueous solutions & spindle speed (RPM) relationship. It is obvious that viscosity range is inversely proportional to the rotational speed. According to Truus et al*.*^51^ alginate viscosity of *Fucus* is comparatively low depending on the gathering time and drying processes of seaweeds, whereas rheological properties of alginate are temperature dependent process. Generally, a reduction in viscosity is often noticed after increasing shear rate, whereas increasing alginate concentrations induced viscosity increments. This rheological behavior demonstrates typical non-Newtonian pseudoplastic pattern on shear thinning characterization in solutions as suggested by Picout et al*.*^52^. This viscosity performance was illustrated by polysaccharides of other algae^53^. The dynamic viscosity manners of alginate polymer are influenced by both polymer structure and mass^54^. Shear thinning pattern of the extracted alginate may be contributed to the hydrodynamic potential resulted throughout the shear decline of alginate structural units^55^. Sutherland^56^ reported that polysaccharide characterized by this rheological pattern can be incorporated into many food industries to modify dynamic viscosity behavior of the present water, changing product texture according to their gel formation ability.

#### Growth responses of *C. vulgaris* to alginate hydrolysate supplementation

**Figure 4:**
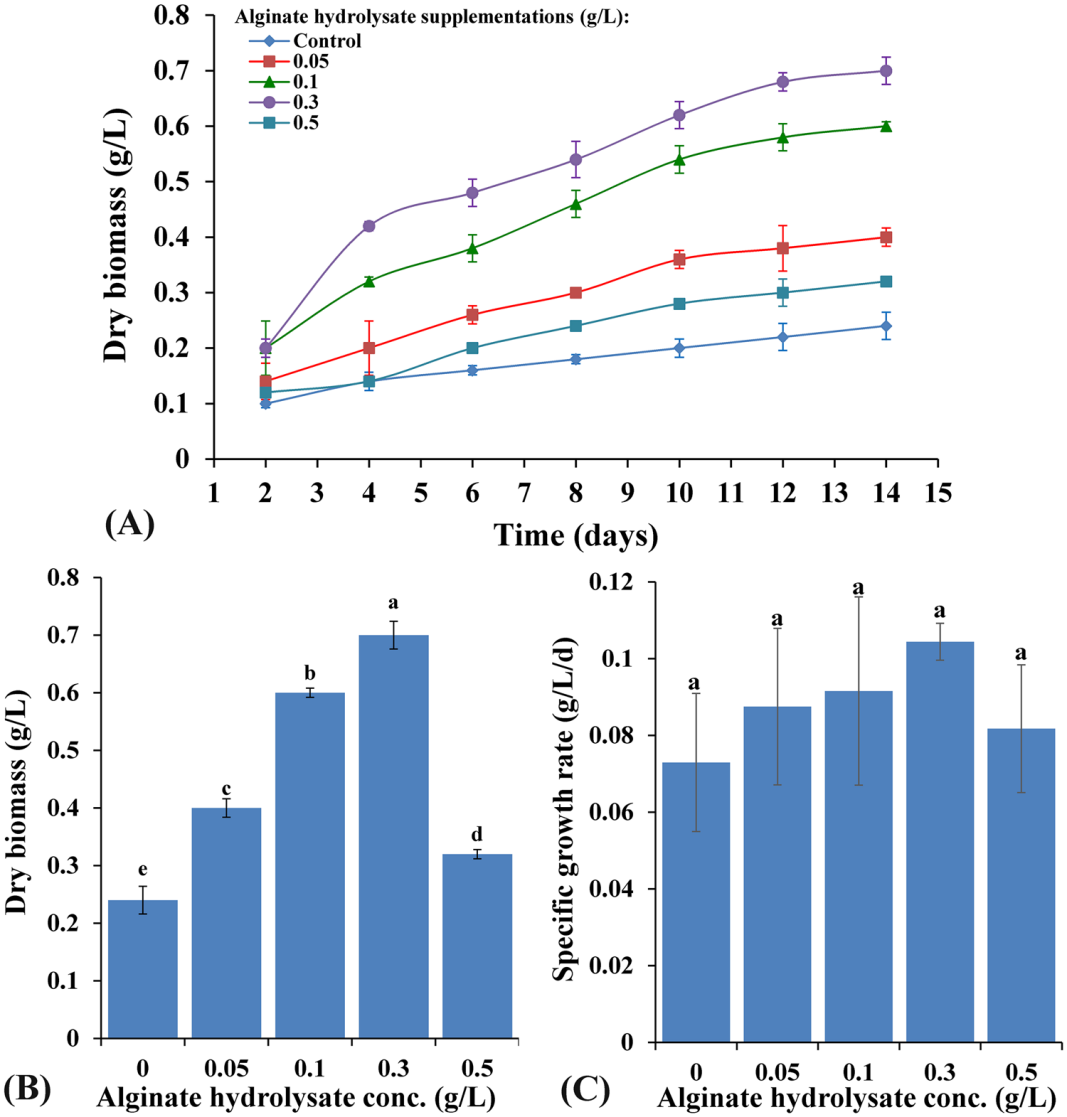
(**A**,**B**) Variation of *C. vulgaris* biomass content (mg/g dry wt) with different alginate hydrolysate supplementations (g/L) and (**C**) Variation of *C. vulgaris* specific growth rate (g/L/d) with different alginate hydrolysate supplementations (g/L).

**Figure 5:**
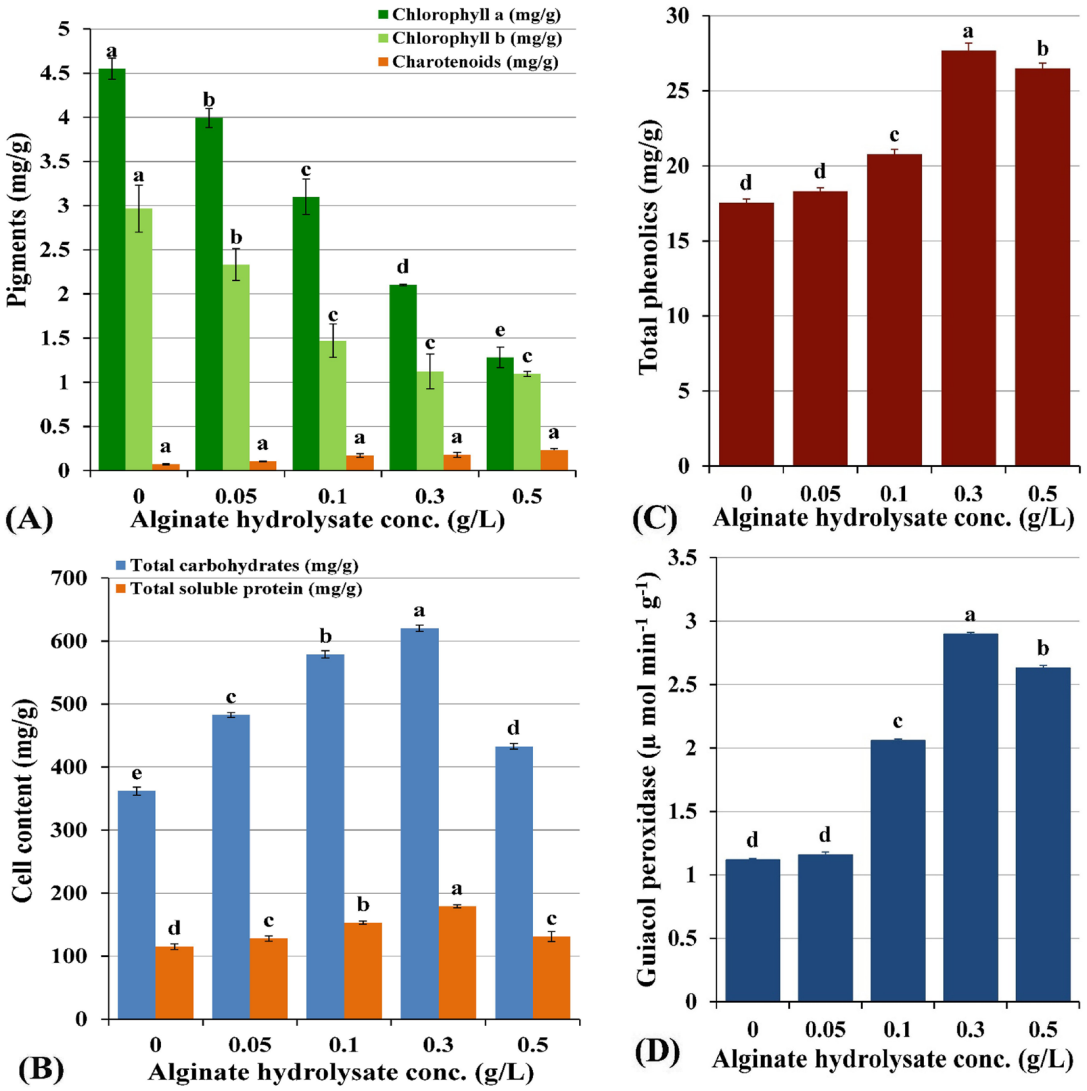
(**A**) Variation of *C. vulgaris* pigments content (mg/g dry wt.), (**B**) Variation of carbohydrates and protein content (mg/g dry wt.) with different alginate hydrolysate supplementations (g/L), (**C**) Variation of *C. vulgaris* phenolics content (mg/g), (**D**) guaiacol peroxidase content (µ mol min^−1^ g^−1^) with different alginate hydrolysate supplementations (mg/L).

Different alginate hydrolysate supplementations (0, 0.05, 0.1, 0.3 and 0.5 g/L) induced various levels of promoting effects on *Chlorella vulgaris* growth and metabolism over 14 days incubation period as can be seen in Fig. 4 A. All the trials were performed in triplicate in order to minimize errors and to calculate standard deviation. As shown in Fig. 4A, C*. vulgaris* growth curve exhibits an exponential phase from the 2nd day till the 12th day after which the growth tends to be in the stationary phase. Significant increments in dry biomass (Fig. 4B), specific growth rate (Fig. 4C), protein and carbohydrate contents (Fig. 5B), total phenolics (Fig. 5C) as well as guiacol peroxidase (Fig. 5D) over the control experiments were documented. On the other hand, all alginate supplementations decreased chlorophyll a and b contents below the control experiment while carotenoids content increased from 0.071 to 0.232 mg/g dry biomass with increasing the alginate supplementation from 0 to 0.5 g/L (Fig. 5A). In general, under this mixotrophic nutritional mode, *Chlorella vulgaris* demonstrated marked increases in most measured growth parameters, whereas, the magnitude of response followed dose response manner. The maximum stimulatory effect was induced by 0.3 g/L alginate hydrolysate supplementation for dry biomass, protein, carbohydrate contents recording values of 0.7 g/L, 179.222 mg/g dry weight and 620.332 mg/g dry biomass; respectively. Higher sodium alginate hydrolysate dose (0.5 g/L) induced nonsignificant increments in the dry biomass, protein and carbohydrate content recording 0.32 g/L, 131.215 mg/g dry weight and 433.143 mg/g dry weight; respectively. On the other hand, the specific growth rate increased in a dose—response manner, taking the bell shape response, achieving the maximum result (0.104 g/L/d) with 0.3 g/L alginate supplementation as illustrated in Fig. 4C.

Figure 5C demonstrated the induced changes in the total phenolic content of *chlorella vulgaris* under this mixotrophic mode of nutrition. Data revealed significant increases in total phenolic contents relative to the control level with increasing alginate hydrolysate concentration (18.315, 20.79, 27.697 and 26.468 mg/g); respectively. Guaiacol peroxidase (GPX) is a key enzyme in regulation of the intracellular H_2_O_2_ equilibrium by transforming H_2_O_2_ into H_2_O along with regeneration of NADP^+^. Data of GPX activity demonstrated significant increases above the control level in concentration depending manner (1.16, 2.059, 2.899 and 2.632 µ mol min^−1^ g^−1^; respectively) as illustrated in Fig. 5D. Thereby, guaiacol peroxidase exhibited significant increases with increasing alginate hydrolysate concentration, displaying maximum activity with 0.3 g/L alginate hydrolysate treatment (2.899 µ Mol min^−1^ g^−1^).

Shen et al*.*^57^ revealed that *Chlorella vulgaris* could grow under different nutritional modes, especially the mixotrophic condition which could induce higher biomass than autotrophic and heterotrophic cultures. Furthermore, substrate concentration and external carbon source, induced final biomass of the mixotrophic cultivations while the lipids content remains unchanged. Many previous studies documented that alginate oligomers have growth promoting effect on higher plants, since *C. vulgaris* is a photosynthetic eukaryotic cell, having structural and functional similarities with higher plant cells so it responds to alginate oligomers in a comparable manner^58^. Yamasaki et al*.*^59^ demonstrated that alginate oligosaccharides may function as growth enhancing agents for certain plant cells and some green algae. In accordance with the present results, Yokose et al*.*^60^ indicated that oligoalginate preparations provided by enzymatic hydrolysis using bacterial alginate lyase, improved growth of *Nannochloropsis oculate.* Whereas, generally, 20 mg/L oligoalginate preparations induced the optimal growth. On the other hand, they found that 40 mg/L slightly decreased the observed growth enhancing activity. As mannuronic acid and guluronic acid are the main components of alginate, it may produce complex with Ca^2+^ and overcome Cu^2+^ induced growth suppression of *N. oculate* ^60^. Diatom *Chaetoceros gracilis* also responded positively to oligoalginate preparation except for diatom genus *Skeletonema* sp., demonstrating a species-specific growth-enhancing significance of the oligo-alginate preparation in microalgae as previously suggested by Yokose et al*.*^60^ . In the present study, alginate hydrolysate concentrations are effective on *C. vulgaris* growth and metabolism in a concentration dependent manner with a bell shaped profile. It can be observed that control experiment (0 g/L alginate hydrolysate) achieved 0.24 g/L biomass whereas the optimum growth (0.7 g/L) was achieved at 0.3 g/L algiante hydrolysate supplementation representing 291.67% of control growth. On the other hand, Ueno and Oda^61^ studied the effect of algiante oligomers different concentations on *Chaetoceros gracilis* (0–1 mg) achieving 140% of control growth when using 0.125 mg/mL alginate oligomers supplementation. On contrast, alginate oligomers have no significant impact on *Skeletonema* sp. growth.

Moreover; 1 mg/L alginate oligosaccharides produced via alginate lyase decomposition, promoted *Chlamydomonas reinhardtii* growth. Moreover; the alginate oligomers produced after acid hydrolysis had no growth–enhancing potentiality as documented by Schafer et al*.*^9^. Also, increments in C16:0, C18:2, and C18:3 fatty acids content of *C. reinhardtii* were noticed after treating with enzyme–decomposing oligo-alginates excluding C18:0 level. On the other hand, acid hydrolysis produced oligo-alginates with no influence on fatty acid content as reported by Schafer et al.^9^. Thus, the oligo-alginate mixture induces the growth of microchlorophytes and increases the content of fatty acids, which may have future prospective in the biotechnological practices for biodiesel production.

According to Naeem et al*.*^62^, the administration of the depolymerized form of irradiated sodium alginate (ISA) as a fertilizing agent resulted in a significant increase in photosynthetic parameters, chlorophyll and carotenoids content as well as growth promotion of *Mentha arvensis*. They supposed that ISA might enable plants to catch more light energy for increasing photosynthesis and/or improving chlorophyll and carotenoids contents consequently after foliar application of ISA as documented by El-Chaghaby et al*.*^63^ previous study. Moreover, when sodium alginate oligomers functioned as plant growth enhancers, they might stimulate plant vegetative growth leading to increased plant productivity relative to control^64^. Degraded alginate (oligosaccharides) induced cell signaling resulted in enhancing different physiological processes in plants^65^. Chlorophyll is one of the cellular compounds on the basis of which microalgal biomass in the culture is estimated and it can be used to measure cell growth. In accordance with our results, Farmer et al*.*^65^ reported that external organic carbon source supplementation may affect photosynthesis and respiration. Also, oligomers produced by alginate depolymerization stimulates the growth and promote the germination and shoot elongation in plants^63^. In accordance with our data, plant growth induction was achieved by the use of radiated sodium alginate oligosaccharides. Gamma irradiation of sodium alginate influences all polymer cross-linking manner, its use affects the biological activities of plant cells^64^.

### Scanning electron microscopy (SEM)

**Figure 6:**
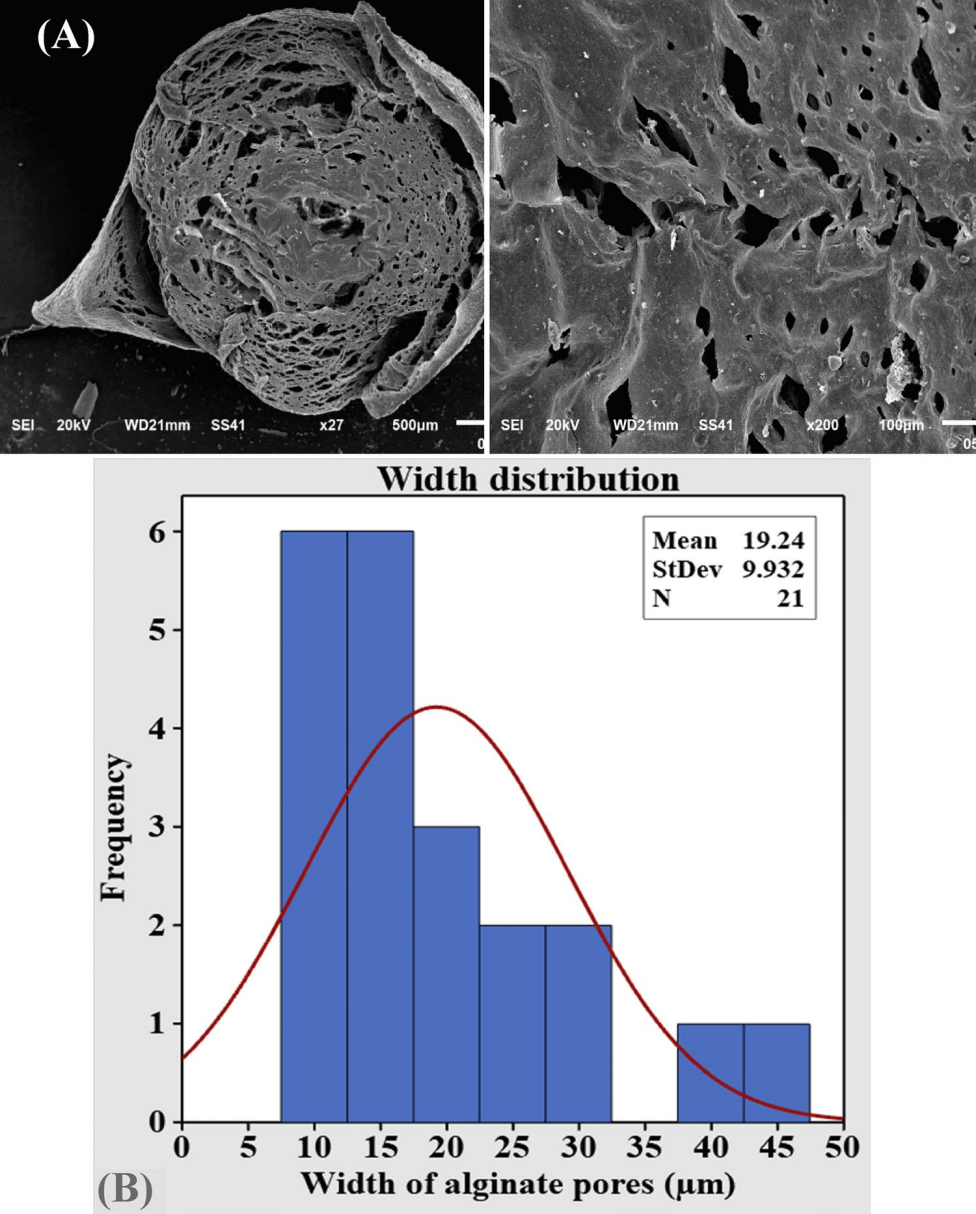
(**A**) SEM electron micrograph of alginate at different magnifications showing alginate pores, (**B**) width ranges of alginate pores versus its frequency,

Alginate as a smart matrix for riboflavin delivery systems was scanned using SEM. The surface and cross-sectional scanning electron microscopy images of the prepared barium alginate beads are illustrated in Fig. 6A. The dry prepared beads were virtually spherical with a mean diameter of 2.37 mm with rough surface. The cross-sectional SEM images demonstrated many closed pores with varying diameters (mean value 19.24 to 166.4 µm) (Fig. 6B). In accordance with the current results, Malakar et al*.*^66^ reported that SEM photographs of liquid paraffin entrapped calcium alginate bead surface showed a rough surface with small pores or channels and no drug crystals were found on the bead's surface, revealing the dispersion of drug crystals in the alginate matrix. Moreover, Rashidzadeh et al*.* ^67^ reported that SEM images of alginate/Ag/Fe_3_O_4_ hydrogel beads are illustrated a clear rough and flat surface.

### FTIR characterization of different sodium alginate bead formulations

FTIR spectra of sodium alginate, Arabic gum, riboflavin, Arabic gum-entrapped barium alginate beads loaded with riboflavin (F1, F2 and F3) and Arabic gum-entrapped barium alginate beads (F4, F5 and F6) are documented in Supplementary Figure S2A,B and Supplementary Table S1. FTIR analysis was applied to investigate the entrapment and stability of R in Alg-AG in the preparations (F1, F2 and F3) with different alginate and Arabic gum concentrations. FTIR profile of sodium alginate (Alg) revealed different chemical functional groups illustrated in Supplementary Fig. S2A. Spectral peaks ranged from 4000 to 3400 cm^−1^ could be assigned to –OH functional group^68^, where FTIR spectrogram of alginate is dominated with strong absorption broad band at 3465 cm^−1^ ascribing hydroxyl group (-OH stretching). The peaks lying in the range of 1060–1030 cm^−1^ (C–O–C stretching) are attributed to the alginate saccharide structure. However, Sinha et al*.*^69^ suggested that guluronic units were recognized at spectral band at 1030 and 1093 cm^−1^ wave number where the mannuronic signals were recognized. The existence of signal peaks around 1628 and 1428 cm^−1^ designating asymmetric and symmetric stretching vibrations of carboxylate anions (COO–)^35^. The spectral band at 2923 cm^−1^ can be attributed to –CH chemical group^37^. FTIR spectrogram of alginate illustrated peak of C–H stretching vibrations recognizing alkanes at 2923 cm^−1^, C=O indicated carbonyl group (amide I band) at 1734 cm^−1^ and COO– stretching vibrations ascribed to carboxylate as well as C–O–C stretching vibrations at 1628 cm^−1^ ^36^. Furthermore, spectral peaks in the range of 848–949 cm^−1^ (C–O) stretching vibration designating the presence of mannuonic and uronic acids; respectively^70^. There are weak interaction between alginate and riboflavin in all alginate-riboflavin mixtures as reported by Aprilliza^36^.

The FTIR profile of alginate and riboflavin–encapsulating alginate Arabic gum composite (Supplementary Fig. S2B) demonstrated some variations in the intensity of peaks especially in the characteristic range 1500 to 400 cm^−1^. These variations recognize the deformations of bonds in the region of carbohydrate. Concerning alginate beads encapsulating riboflavin, (Supplementary Fig. S2B) showed characteristic broad bands in the range of 4000 to 3100 cm^−1^ designating stretching vibration of O–H group as well as the C = C aromatic group of both alginate riboflavin, in addition to presence of C = O ketonic group of riboflavin ^68^ which designate stability of riboflavin in the formed beads.

According to Hosseini et al*.*^71^, the characteristic chemical groups of riboflavin are demonstrated in lower intensities and may be obscured by other signals confirming the existence of electrostatic interactions between the blend components. FTIR charts of Arabic gum-entrapped barium alginate beads loaded with riboflavin exhibited comparable features indicating absence of interference between R and Alg-AG ingredients besides non-participation of the ketonic carbonyl in coordination with alginate binding centers^72^.

For Alg-AG-R physical interaction characterization, the R carbonyl stretching region (1730–1620 cm^−1^) was analyzed. The present data illustrated that characteristic acid carbonyl stretching band of the pure drug appeared unchanged in the polymer/drug physical mixtures, and the spectra seemed to be the sum of the spectra of the pure components.

### Riboflavin encapsulation efficiency of the prepared riboflavin alginate-Arabic gum beads

**Figure 7:**
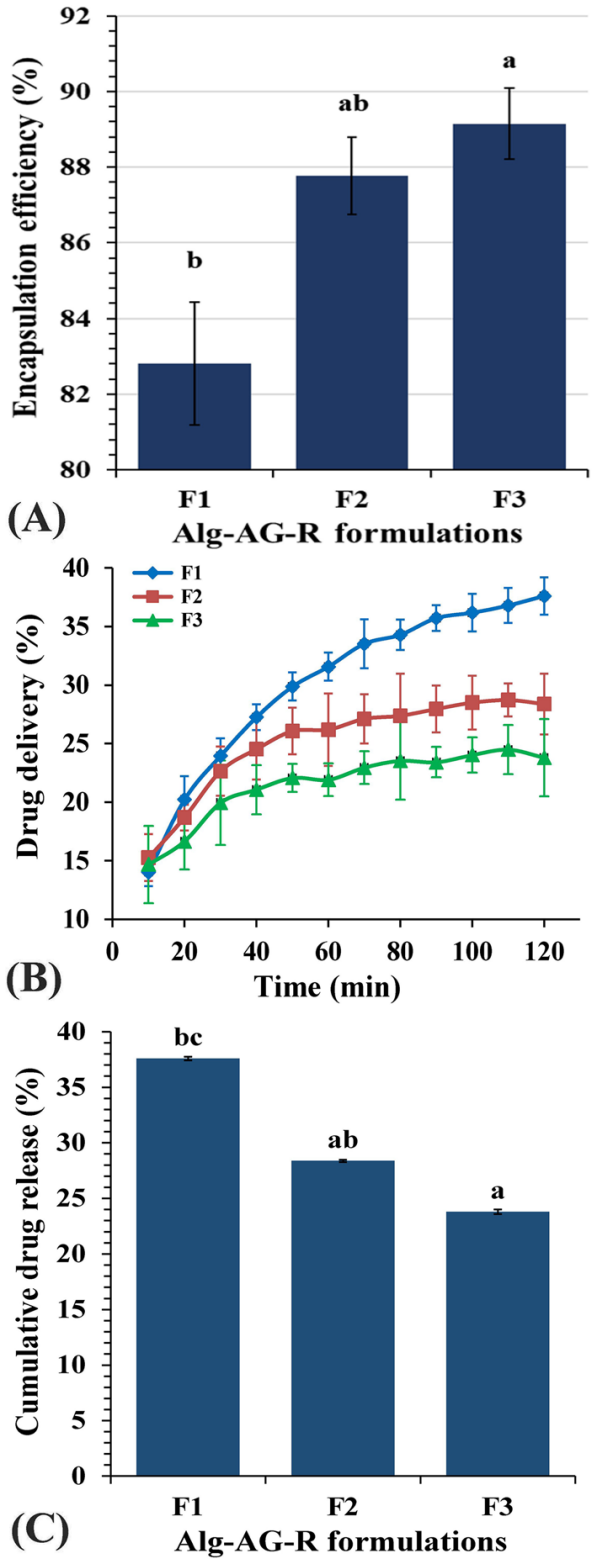
(**A**) Encapsulation efficiency of riboflavin alginate-Arabic gum formulations, (**B**) In vitro riboflavin release from riboflavin entrapped Alg-AG beads, (**C**) Cumulative drug release percent.

Sodium alginate has the ability to form rigid gels with divalent cations. Although it is relatively easy to describe alginates in terms of M and G units, the detailed molecular compositions of alginates in terms of block lengths and block distributions are more difficult to determine. The formation of riboflavin-entrapped barium alginate beads is a simple and significant process for drug delivery. Riboflavin encapsulation efficiency of the prepared riboflavin alginate-Arabic gum beads ranged from 82.81 to 89.15% (Table 1, Fig. 7A) according to the formulation composition of the beads. The highest encapsulation efficiency was positively correlated to the Arabic gum concentration which was observed in Alg-AG-R 0.2 formulation. This pattern of response could be attributed to the partitioning of certain amounts of riboflavin in the Arabic gum phase and/or constitution of an Arabic gum barrier that prevents external passage of riboflavin molecules throughout preparation. Whereas, the physical interaction and/or enlargement of the complicated cross-linked barium alginate network may facilitate the entrapment of the drug (riboflavin) according to the interpretation of Malakar et al*.*^66^. Results of Azad et al*.*^73^ are in line with the current results whereas, they documented that the EE% of black seed oil in alginate beads ranged from 67.20 to 104.50% and increased with increasing voltage and flow rate. The encapsulation efficiency was found to be dependent on the encapsulating matrix's strength^74^. On the other hand, the cross-linking ability of alginate may be able to increase the encapsulation efficiency of oil in alginate^75^.

**Table 1:** In vitro riboflavin release encapsulation efficiency and kinetic parameters.

Alginate bead Formulations	EE (%)	Kinetic parameters								
		Zero-order kinetics		1st-order kinetics		Higuchi model		Korsmeyer-Peppas model		
		K_0_	R^2^	K_1st_	R^2^	K_H_	R^2^	K_p_	R^2^	n
F1	82.81	4 × 10^–7^	0.882	4 × 10^–7^	0.882	5 × 10^–6^	0.962	11 × 10^–6^	0.978	0.389
F2	87.76	2 × 10^–7^	0.777	10^–7^	0.792	2 × 10^–6^	0.895	16 × 10^–6^	0.943	0.251
F3	89.15	10^–7^	0.791	2 × 10^–7^	0.777	3 × 10^–6^	0.887	17 × 10^–6^	0.948	0.204

### In vitro release studies

The in vitro prolonged, sustained release profile (Fig. 7B) of Alg-AG-R with different alginate-Arabic Gum composite-riboflavin formulations could be designated as two-step biphasic process. Concerning Alg-AG-R 0.1, the first phase continued for 70 min which designated by burst release, followed by a stationary phase, whereas in Alg-AG-R 0.15 and Alg-AG-R 0.2 formulations the exponential R release continued for 50 min only then a stationary one operated to the end of the experiment. Results indicate that Alg-AG-R 0.1 formulation exhibits the highest R release. After 2 h, Fig. 7C showed different patterns of cumulative drug release (%) profiles of riboflavin with the following hierarchy Alg-AG-R 0.1 (37.598%) > Alg-AG-R 0.15 (28.375%) > Agl-AG-R 0.2 (23.802%). Increments in R release from Alg-AG-R was parallel to increasing alginate content and to decreasing Arabic gum content in the beads on the other side.

The current results indicated that riboflavin release rate significantly increased with decreasing Arabic gum incorporated concentration i.e., by the relative increasing alginate concentration. Owing to interpretation of Peppas and Narasimhan^76^ , drug molecules dissolution from polymeric blends depends generally on two key phenomena. Firstly, decomposing of the encapsulating substance and dispersion of the drug molecules through the polymeric matrix; however, the second phenomenon concerned with swelling, chemical decomposition, osmotic consequences. Based on Bera et al*.*^77^ interpretations, slow prolonged release could be ultimately due to the residual drug that is dispersed in Arabic gum pockets of the beads formulating a drug-Arabic gum dispersed matrix. Consequently, R delivery from Alg-AG-R beads to the dissociation medium may exhibit two steps, including the diffusion of the drug out of Arabic gum pockets into the barium alginate matrix at first, then it could be distributed outwards barium alginate matrix into the dissociation medium. Another point of view was suggested by Bera et al*.*^77^ who described the behavior of R release to additional Arabic gum barrier formation.

Beirão-da-Costa et al*.*^78^ documented the importance of alginate volume expansion in the initial exponential phase of R release from Alg-AG matrix. Helrich^79^ suggested that drug release from microparticles might be occurred by different modes of action comprising surface erosion, disintegration, diffusion and desorption. According to Anitha et al*.*^80^, the initial fast release rate could be attributed to riboflavin molecules adsorption onto and near the particle surface where the alginate dissolution rate is high. Hosseini et al*.*^81^ and Siepmann et al*.*^82^ documented an alternative interpretation, that slow R delivery during second stage might be attributed to the continuous diffusion of R into Alg-AG microbeads with time, in addition to the maintenance of almost-linear R concentration gradients over extended periods within the Alg-AG microbeads. Alginate microparticles were considered safe, Abdelaziz et al*.*^83^ reported alginate nanoparticles as a safe delivery system for miltefosine in the treatment of candidiasis and cryptococcosis. Alginate nanoparticles toxicity were assessed on red blood cells and *Galleria mellonella* larvae. Miltefosine in alginate nanoparticles existed neither hemolytic effect, nor, toxicity in larvae. Results showed the nontoxic use of alginate-based drug-delivery systems as carriers to control the fungal infection in the in vivo model of *G. mellonella*^19^*.* Shanmuganathan et al*.*^84^ found that various drugs and nanoparticles have been encapsulated or adhered over chitosan nanoparticles and applied for cancer treatment.

### Kinetic studies

The mathematical kinetic models: zero order (Supplementary Fig. S3A), first order (Supplementary Fig. S3B), Higuchi (Supplementary Fig. S3C) and Korsmeyer-Peppas (Supplementary Fig. S3D) models were followed for evaluating the in vitro riboflavin release. The zero-order kinetic model is a relation between time (min) and log cumulative percent of drug release, achieving R^2^ values ranging from 0.8823 to 0.7914. The first order kinetic model is a relation between time (min) and Ln(1-F), giving R^2^ values ranging from 0.777 to 0.7915. Higuchi model is a relation between t_1/2_ and log cumulative percent of drug release achieving R^2^ results range of 0.9616 to 0.8945. Korsmeyer Peppas model is a relation between Ln t and log cumulative percent of drug release giving R^2^ results range of 0.9783 to 0.9484. The resulted kinetic parameters of curve fitting in the previously mentioned mathematical models are listed in Table 1. Investigating data of corresponding correlation coefficients (R^2^) of Alg-AG-R microbeads in the dissociation medium indicated that riboflavin release follows Higuchi model (R^2^ = 0.962–0.887) and Korsmeyer Peppas model (R^2^ = 0.948–0.978) over a period of 2 h.

Different riboflavin preparations release behavior was assessed using Korsmeyer–Peppas model (Supplementary Fig. S3D) which differentiate between different release mechanisms: Fickian release (controlled release), non-Fickian release (irregular transport), and case-II transport (relaxation-controlled release). Value of n ≤ 0.43 designates Fickian release, n value range between 0.43 and 0.85 indicates non-Fickian release, Whereas, n ≥ 0.85, it is case-II transport which includes polymer disbanding and enlargement of polymeric chain^85^. The present data indicate Fickian release (diffusion-controlled release) (n ranges from 0.204 to 0.389) as reported by Siepmann et al*.*^82^ . In accordance with our results, Azad et al*.*^73^ reports that the black seed oil release from alginate fitted with Korsemeyer–Peppas kinetic model (R^2^ = 0.900 to 0.997) over two h period, except for the release exponent (*n*) values which were in the range of 0.49 to 0.61, designating non-Fickian diffusion in acidic media (pH 1.2). According to the interpretation of Danarto et al*.*^86^, the ability to encapsulate riboflavin in the alginate—gum Arabic hydrogel could be contributed to the development of an "eggbox" structure during the crosslinking process with Ba^2+^ ions. They suggested that higher sodium-alginate concentration will result in more loading of riboflavin. This might be attributed to the number of "egg-box" structures that are formed within the alginate molecule whereas 3% Na-alginate concentration was the optimum for the loading process.

## Materials and methods

### Collection and preparation of macroalgal sample

*Sargassum latifolium* (Turner), C. Agardah was collected from the shores of Safaga-Quaser, Red Sea Governorate, Egypt, on June 2017, following the institutional, national and international guidelines and legislation. The macroalga was kindly identified by prof. Dr. Mohamed S. Abdel-Kareem, Botany and Microbiology Department, Faculty of Science, Alexandria University, Egypt. The voucher specimen (*Sargassum latifolium*—Herb. Nasr—1ph-2021) has been deposited at the herbarium of late professor Abdel-Halim Nasr at Botany and Microbiology Department, Faculty of Science, Alexandria University, Egypt. *Sargassum latifolium* thalli were surface cleaned using distilled water, then dried at 60 °C until reaching constant weight. Thereafter, thalli were crushed into small pieces (0.1–0.5 cm)^87^.

### Extraction of sodium alginate

Alginate extraction was conducted following Bouissil et al*.*^24^ protocol with some modifications. 20 g crushed algal biomass was washed with 300 mL boiling distilled water for 30 min. Then the algal biomass was boiled with 300 mL of 0.5% CaCl_2_ solution for 30 min. Afterward, algal residue was obtained and boiled with 300 mL of 0.5% NaCl for 1 h. After filtration the algal sample was boiled with 100 mL of Na_2_CO_3_ (5%) for 30 min with intensive stirring. After filtration, sodium alginate was precipitated by dehydration using 80% ethyl alcohol and then dried at 50 °C and crushed before storage. Alginate yield was presented as a weight percentage of algal dry biomass according to Belattmania et al*.*^22^.

#### Chemical composition

Total carbohydrates content was estimated according to the method of Farmer et al*.*^65^ whereas soluble protein was determined following Idrees et al*.*^88^ protocol. Lipid content was assessed gravimetrically using chloroform–methanol system following the protocol of Currie and Turvey^89^. Sulfate content was assessed after hydrolysis of alginate with 2 M HCl for 2 h at 100 °C according to Association et al*.*^90^. Uronic acid content was evaluated following the protocol of Blumenkrantz et al*.*^91^. An alginate specimen was added to a solution of sodium tetraborate in concentrated H_2_SO_4_, followed by addition of m-hydroxydiphenyl reagent with shaking. The indicative color was developed within 5 min and measured spectrophotometrically at 520 nm.

#### UV–VIS spectroscopy

The UV–Vis absorbance spectrum of *Sargassum latifolium-*derived alginate solution was recorded using ATI UNICAM-UV/Visible spectrophotometer vision software V3.20 – England in the range of 200–800 nm in the Unit of Spectra –Faculty of Sciences – Mansoura University—Egypt.

#### ^1^H NMR spectroscopy

^1^H NMR spectroscopy is a good method for detecting the composition, beside reviewing the block structure of the extracted alginate^22^. Alginate sample solution (6 mg/mL Deuterium water) was used for ^1^H NMR analysis using ECA 500 II (JEOL—Japan) NMR spectrophotometer in the NMR Unit—Faculty of Sciences—Mansoura University—Egypt. The ^1^H NMR results were measured using field strength 500 MHz.

#### Fourier-transform infrared (FTIR) spectroscopy

Dried alginate sample (1 mg) was dispersed and pressed in 0.1 g anhydrous potassium bromide. Spectra of IR were documented at room temperature in the frequency range 400–4000 cm^−1^ ^92^ using Mattson 5000—Japan FTIR spectrometer in the Unit of spectra—Faculty of Sciences—Mansoura University—Egypt .

### Rheological measurements

Rheological characterization of the alginate extract was performed in the concentrations of 5, 10 and 15 mg/mL using the BROOKFIELDDV-3-USA Ultra Programmable Rheometer in the physics department—Faculty of Science—Mansoura university—Egypt that measures fluid parameters of shear stress and viscosity at given shear rates at 25°C^93^. Shear stress and viscosity were documented as a function of practical shear rate (10–500 s^−1^). All rheological data were achieved in triplicate and the mean of values were calculated.

### X-ray diffraction (XRD)

The X-ray diffraction data of sodium alginate were measured using SHIMADZU-6000—Japan Diffractometer in The Egyptian Atomic Energy Authority. Measurement conditions were, the X-ray tube with Cu anode target, a voltage of 40 kV, and a current of 30 mA. XRD diffractograms were recorded in the 2θ range of 4.0–90 in continuous scan mode with speed of 8.0 deg/min.

### Thermogravimetric analysis (TGA)

Thermogravimetry (TG), is an analysis by which the change in a specimen mass exposed to progressive heating with a constant rate is documented and illustrated vs. temperature, is an efficient protocol for investigating the thermal stability of a compound. TGA of sodium alginate was performed using a TGA-50 SHIMADZU—Japan Thermogravimetric analyzer at Egyptian Atomic Authority. The process was conducted in the temperature range 20–800ºC at a heating rate of 10ºC min^−1^ in nitrogen atmosphere. Data were expressed as percent of mass loss (Δ mass %) versus temperature.

### Growth responses of *C. vulgaris* to alginate hydrolysate supplementation

Alginate hydrolysate was prepared according to the protocol of Hotchkiss et al.^94^, with some modifications. Sodium alginate solution (1%) was acidified with 2 mL of 0.2 N HCl and autoclaved at 121ºC for 20 min. Then cooled and neutralized using sodium carbonate. Finally, a homologous extract of oligo-guluronic acid was obtained.

*Chlorella vulgaris,* the test microalga, was provided from the microalgal collection of Phycology Laboratory-Faculty of Science—Mansoura University. *C. vulgaris* was grown up in axenic cultures and incubated for 14 days at 25 ± 2ºC under constant illumination (59.4 µmol s^−1^ m^−2^) using BG11 media^93^. *C. vulgaris* was grown under a mixotrophic mode of nutrition using alginate hydrolysate as an external carbon source in the following concentrations (0.05, 0.1, 0.3 and 0.5 g/L) to investigate *C. vulgaris* growth and metabolism. Dry biomass was estimated at two days intervals. Moreover, at the end of the experiment, light harvesting pigments, protein as well as carbohydrate contents were estimated as previously mentioned. Photosynthetic pigments (chlorophylls and carotenoids) were assessed following the method of Metzner et al*.*^95^. Soluble phenols content was assessed according to the method of Gillespie et al*.*^96^, guaiacol peroxidase (GPX) activity was estimated following the protocol of Curtis et al*.*^97^.

### Sodium alginate as a carrier matrix for riboflavin drug delivery in simulated gastric juice

For preparing Arabic gum-entrapped barium alginate beads loaded with riboflavin (Alg-AG-R), the emulsion-gelation method of Lin et al*.*^98^ was followed. Two grams of sodium alginate were dissolved in 100 mL deionized water with stirring. Liquid Arabic gum (0.1, 0.15 and 0.2 g) and riboflavin (5 mg) were mixed with the previously prepared sodium alginate solutions, then aqueous preparations of sodium alginate and liquid Arabic gum with riboflavin were stirred for 30 min at 150 rpm for emulsion stability. For producing spherical beads, the formed polymeric emulsion was dropped through glass syringe with a size-22 disposable needle into BaCl_2_ solution (1 g BaCl_2_ in 100 mL of 10% acetic acid) with continuous stirring. For strengthening the beads, they were permitted to stand for 15 min in the BaCl_2_ solution, then filtered and washed twice with deionized water. The resulted beads formulations are Alg-AG-R 0.1 (F1), Alg-AG-R 0.15 (F2), Alg-AG-R 0.2 (F3), Alg-AG 0.1 (F4), Alg-AG 0.15 (F5) and Alg-AG 0.2 (F6), illustrated in Table 2.

**Table 2:** Various Alginate- Arabic gum formulations with and without riboflavin.

Formulation	Alginate (g/100 mL)	Arabic gum (g/100 mL)	Riboflavin (mg/100 mL)	Abbreviation
F1	2	0.1	5	Alg-AG-R 0.1
F2	2	0.15	5	Alg-AG-R 0.15
F3	2	0.2	5	Alg-AG-R 0.2
F4	2	0.1	–	Alg-AG 0.1
F5	2	0.15	–	Alg-AG 0.15
F6	2	0.2	–	Alg-AG 0.2

The simulated gastric fluid was prepared by dissolving 3.5 g glucose, 2.05 g NaCl, 0.60 g KH_2_PO_4_, 0.11 g CaCl_2_, 0.37 KCl in 200 mL deionized water. The pH of the solution was adjusted to 2 using 1 M HCl and to final volume of 1 L.

#### Scanning electron microscopy (SEM)

The morphology and surface of the alginate beads were assessed using scanning electron microscope (JEOL JSM 6510/V, Japan) at the Electron Microscope Unit, Mansoura University—Egypt.

### In vitro release studies of riboflavin

The in vitro release of riboflavin drug from different liquid Arabic gum-entrapped barium alginate beads formulations was investigated for 120 min with regular time intervals. Different Arabic gum—entrapped barium alginate beads formulations loaded with riboflavin were added to the simulated gastric fluid with stirring (50 rpm) at 35 ± 2 °C. For investigating riboflavin release, sampling solution aliquots at 10 min intervals were used to estimate riboflavin concentration specrophotometricaly at 444 nm using riboflavin standard curve. Encapsulation efficiency percent (EE %) was determined following the equation of Nallasamy et al*.*^99^.

1 $$  {\text{EE}}\left( \%  \right) = {\text{total}}\;{\text{amount}}\;{\text{of}}\;{\text{loaded}}\;{\text{R}}/{\text{initial}}\;{\text{amount}}\;{\text{of}}\;{\text{R}} \times {\text{1}}00  $$

### Riboflavin release kinetics

For interpreting the performance of riboflavin release from the barium alginate beads in simulated gastric fluid (pH 2), suitable mathematical models must be followed. In vitro riboflavin release from different alginate-Arabic gum beads loaded with riboflavin (Alg-AG-R) data were assessed kinetically through different mathematical models: (Zero-order, First-order, Higuchi and Korsmeyer-Peppas model)^100^ as shown in Table 3; where F represents the drug fraction released at time t, K_0_ represents zero-order release constant, K_1_ represents the first-order release constant, K_H_ represents Higuchi dissolution constant and K_p_ is Korsmeyer-Peppas constant and n represents the release exponent.

**Table 3:** Kinetic models applied on riboflavin release from R-Alg-AG beads.

Model	Formula	Constant
Zero-order model	F = K_0_t	K_0_
First-order model	Ln (1 − F) = − K_1st_ t	K_1st_
Higuchi Model	F = K_H_t^1/2^	K_H_
Korsmeyer-Peppas model	F = K_p_t^n^	K_p_

### Statistical analysis

Data were subjected to statistical analysis following Zobel et al.^101^ using one-way analysis of variance followed by least significant difference (LSD) analysis, *P*-values more than 0.05 were considered statistically, non-significant, whereas P-value less than 0.05 represent statistically significant data. Results were expressed as mean ± standard deviation.

## Conclusion

In this study, *Sargassum latifolium*—derived alginate hydrolysate can be used as *C. vulgaris* growth bio-stimulant. A new sustained release system of riboflavin-entrapped barium alginate-Arabic gum drug delivery matrix was elucidated. Moreover, drug entrapment technique using alginate is a valuable developmental tool of the multi particulate system for drug delivery even for a highly water-soluble drug such as riboflavin.

## Supplementary Information

Supplementary Information.
