# Supplementary material for: Characterization of alginate extracted from Sargassum latifolium and its use in Chlorella vulgaris growth promotion and riboflavin drug delivery
Source: Sci Rep. 2021 Aug 18;11:16741. doi: 10.1038/s41598-021-96202-0 (PMC8373903; doi:10.1038/s41598-021-96202-0)
Supplement: Supplementary file 1 — Supplementary Information. [file 41598_2021_96202_MOESM1_ESM.doc]

**Characterization of alginate extracted from *Sargassum latifolium* and its use in *Chlorella vulgaris* growth promotion and riboflavin drug delivery**

**^1^Shimaa R. Dalal, ^1^Mervat H. Hussein, ^2^Noura El-Ahmady El-Naggar*, ^3^Sahar I. Mostafa, ^1^Sami A. Shaaban-Dessuuki**

^1^Botany Department, Faculty of Science, Mansoura University, Mansoura, Egypt

^2^Department of Bioprocess Development, Genetic Engineering and Biotechnology Research Institute, City of Scientific Research and Technological Applications (SRTA-City), Alexandria 21934, Egypt

^3^Chemistry Department, Faculty of Science, Mansoura University, Mansoura, Egypt

*To whom correspondence should be addressed.

**Dr. Noura El-Ahmady Ali El-Naggar**

**Address:**

Bioprocess Development Department,

Genetic Engineering and Biotechnology Research Institute,

City of Scientific Research and Technological Applications,

New Borg El- Arab City, 21934, Alexandria, Egypt

**Tel:** (002)01003738444

**Fax:** (002)03 4593423

**E-mail:** [nouraelahmady@yahoo.com](mailto:nouraelahmady@yahoo.com)

**Supplementary Table S1: FTIR spectral analysis of different bead formulations, alginate, Arabic gum and riboflavin**

| **Assignment** | **Wave number (cm^−1^)** | | | | | | | | |
| --- | --- | --- | --- | --- | --- | --- | --- | --- | --- |
| Samples | F1 | F2 | F3 | F4 | F5 | F6 | Alg | AG | R |
| O-H stretching, N-H stretching | 3480 | 3676 | 3677 | 3448 | 3449 | 3447 | 3465 | 3450 | 3494 |
| =C–H stretching | 3000 | – | – | – | – | – | – | – | 3210 |
| C–H stretching | 2927 | 2927 | 2926 | 2926 | 2927 | 2926 | 2923 | 2926 | 2935 |
| Asymmetrical (C–H) CH_2_, aromatic and/or vinylic C–H stretching,  (CH)-anomer stretching | 2927 | 2927 | 2926 | 2926 | 2927 | 2926 | 2923 | 2926 | – |
| Symmetrical (C–H) CH_2_ (Aliphatic C–H) stretching, | 2857 | – | – | – | – | – | 2854 | – | – |
| Aliphatic C=O stretching, Aromatic C=O stretching | – | – | 1773 | – | – | – | 1734 | – | 1732 |
| COO− asymmetric stretching | 1630 | 1628 | 1629 | 1632 | 1634 | 1628 | 1628 |  |  |
| C=C stretching | – | – | 1523 | 1419 | 1420 | 1420 | 1465 | 1457 | 1504 |
| COO− symmetric stretching | – | – | – | – | – | – | – | 1457 | 1460 |
| C–O stretching, C–H deformation, C–O–H deformation | 1259 | 1257 | 1263 | 1266 | 1269 | 1266 | – | – | 1247 |
| C-C stretching, C–X stretching | 1084 | 1099 | 1097 | 1030 | 1030 | 1029 | 1087 | 1030 | 1276 |
| -C-O, C=S stretch | – | – | 1097 | 1088 | 1087 | 1085 | 1087 | – | 1075 |
| C-O-C stretching, S=O stretch | – | 1056 | – | 1030 | 1030 | 1029 | – | 1030 | – |
| C-O-S stretching | 814 | 855 | 813 | 875 | 876 | 875 | 848 | 882 | 815 |
| C=O stretching secondary amide | 721 | 703 | 671 | 673 | 674 | 713 | – | 669 | 677 |
| C-X stretching | 404 | 554 | 526 | 422 | 404 | 520 | 601 | 604 | 448 |


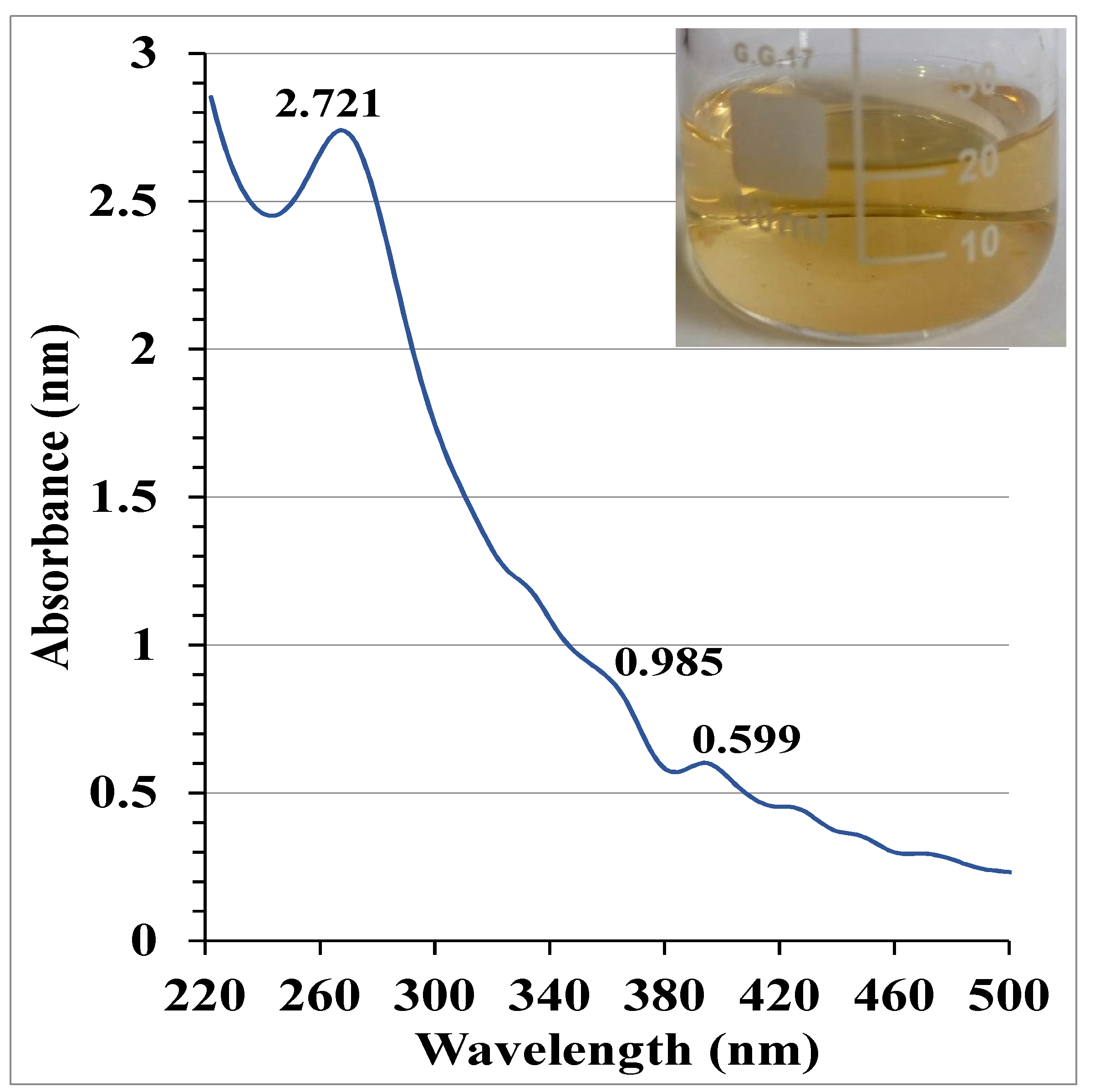


**Supplementary Figure S1**: UV-absorbance spectrum of extracted sodium alginate.


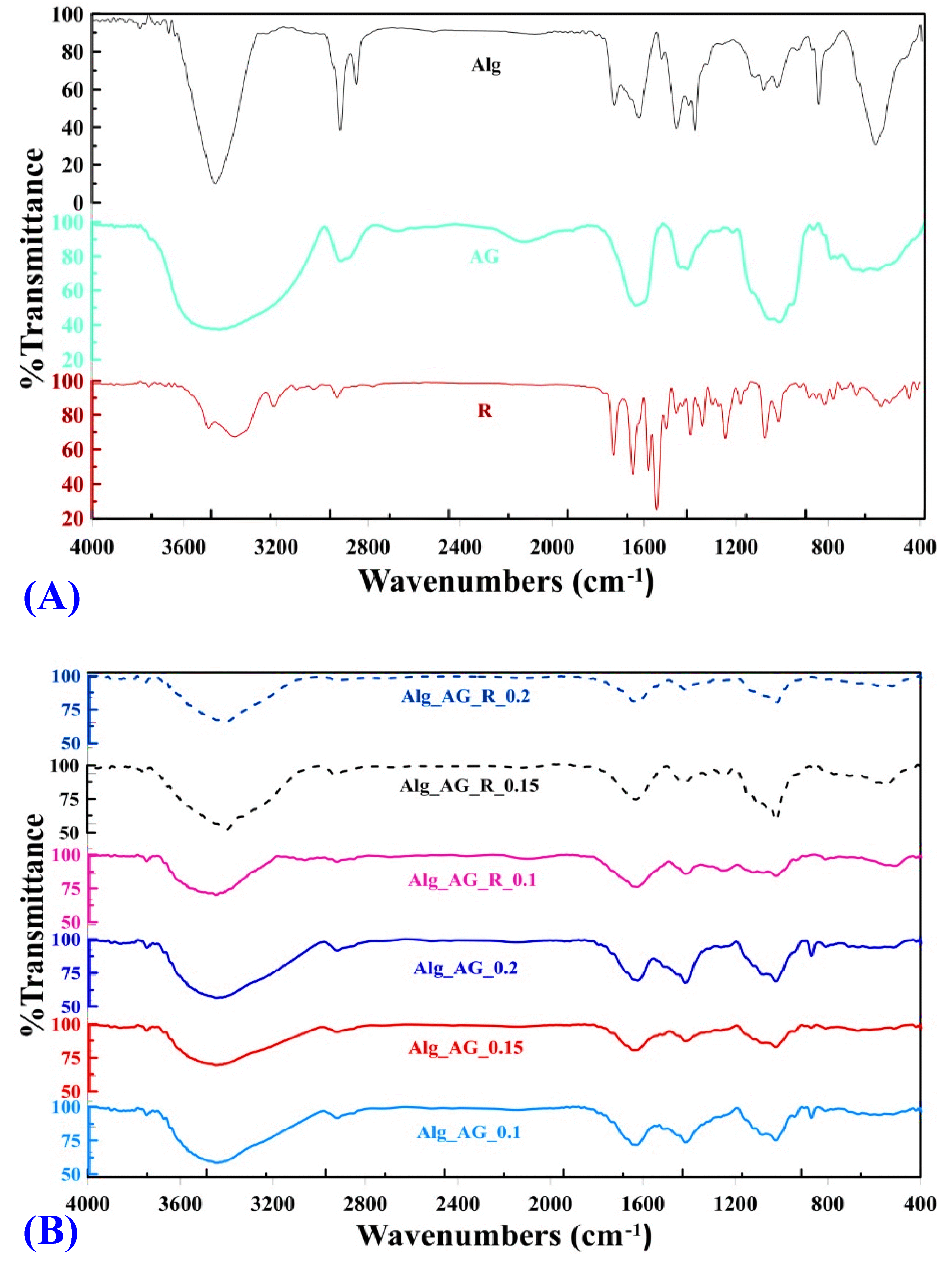


**Supplementary Figure S2 A)** FTIR spectral analysis of sodium alginate, Arabic gum and riboflavin, **B**) FTIR characterization of different alginate formulations.


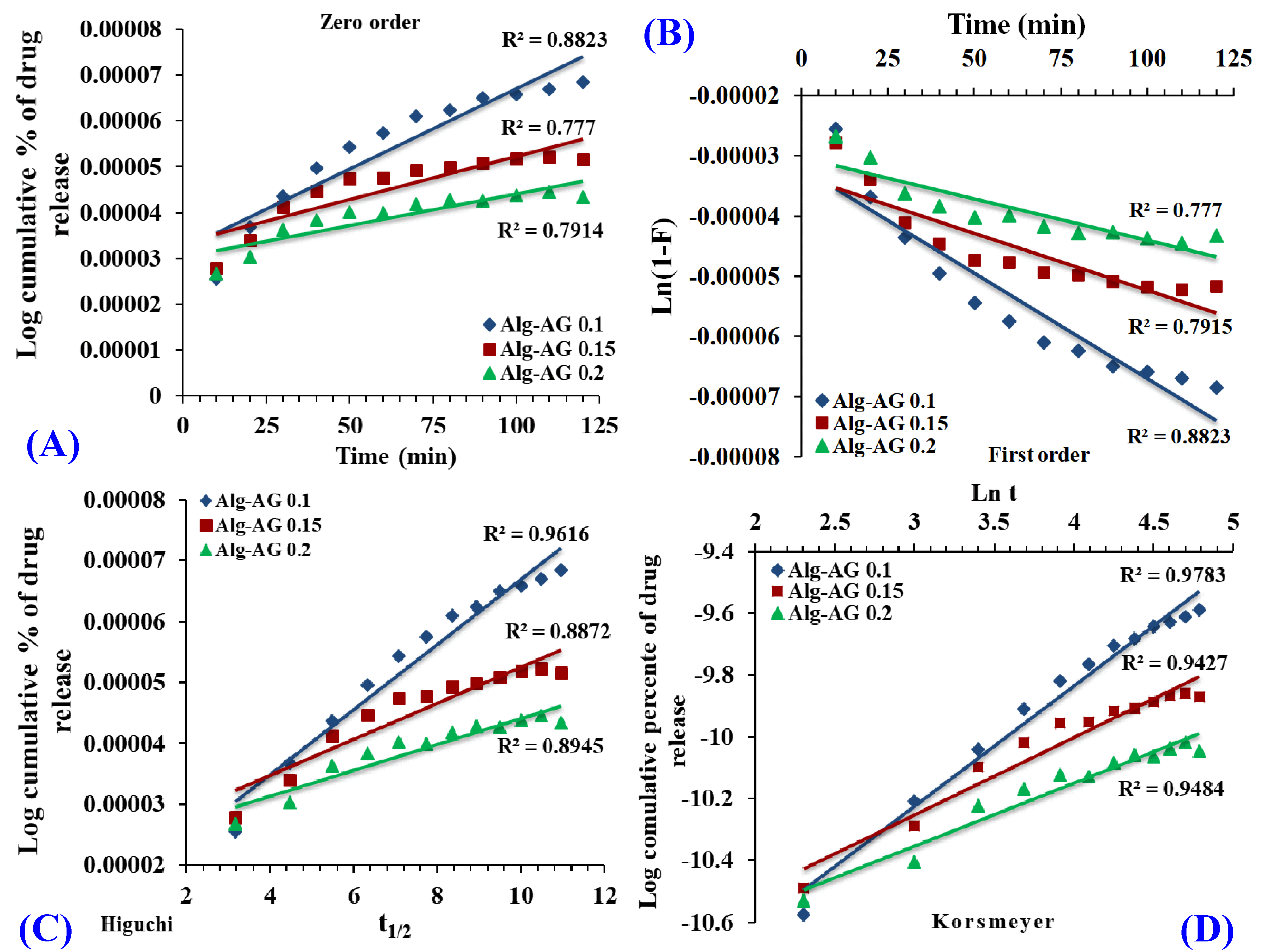


**Supplementary Figure S3** **A**) Zero order kinetic model for different Alg-AG formulations, **B**) First order kinetic model for different Alg-AG formulations, **C**) Higuchi kinetic model for different Alg-AG formulations, **D**) Korsmeyer-Peppas kinetic model for different Alg-AG formulations.

**Results and discussion:**

Our results do not fit zero order kinetic model (R^2^ ranged from 0.791 to 0.882) and first order kinetic model (R^2^ ranged from 0.777 to 0.882), so it is normal that their data does not fit a linear curve. On the other hand, Supplementary Figure S3 C (Higuchi kinetic model) and Supplementary Figure S3 D (Korsmeyer-Peppas kinetic model) have only few points that are out of the linear curve and their values of R^2^ are in the range of 0.9, revealing the accuracy and fitting with Higuchi and Korsmeyer-Peppas models. Moreover; the study of Soni and Yadav^1^ was in accordance with data of the present manuscript. They reported that their data fitted with Higuchi and Korsmeyer-Peppas kinetic models according to R^2^ values that were in the range of 0.9 although some of the points are out of the linear curve.

**References:**

1. Soni, G. & Yadav, K. S. High encapsulation efficiency of poloxamer-based injectable thermoresponsive hydrogels of etoposide*. Pharm. Dev. Techno*l**.** 19, 651–661 (2014).
